# Exogenous α-lipoic acid mitigates lead (Pb) toxicity in tomato seedlings by regulating metabolites, nutrient uptake, antioxidant defense and redox balance maintenance

**DOI:** 10.3389/fpls.2025.1611383

**Published:** 2025-09-24

**Authors:** Khaled M. A. Ramadan, Sallah A. Al Hashedi, Adil AlShoaibi, Muhammad Naeem Sattar, Doaa Bahaa Eldin Darwish, Khulud S. Albalawi, Fahad Mohammed Alzuaibr, Eslam S.A. Bendary, Hala B. Khalil, Hesham S. Ghazzawy, Mahmoud Adel Ahmed Ali, Mohamed F. M. Ibrahim

**Affiliations:** ^1^ Central Laboratories, Department of Chemistry, King Faisal University, Al-Ahsa, Saudi Arabia; ^2^ Physics Department, College of Science, King Faisal University, Al-Ahsa, Saudi Arabia; ^3^ Department of Biology, Faculty of Science, University of Tabuk, Tabuk, Saudi Arabia; ^4^ Department of Agricultural Biochemistry, Faculty of Agriculture, Ain Shams University, Hadayek Shobra, Cairo, Egypt; ^5^ Biological Sciences Department, College of Science, King Faisal University, Al-Ahsa, Saudi Arabia; ^6^ Date Palm Research Center of Excellence, King Faisal University, Al-Ahsa, Saudi Arabia; ^7^ Horticultural Department, Faculty of Agriculture, Ain Shams University, Hadayek Shobra, Cairo, Egypt; ^8^ Department of Agricultural Botany, Faculty of Agriculture, Ain Shams University, Cairo, Egypt

**Keywords:** *Solanum lycopersicum* L., water contamination, heavy metals, lead toxicity, ascorbate–glutathione cycle, reactive oxygen species (ROS) and redox balance

## Abstract

Water contamination with heavy metals drastically affects plant growth and development. It is more dangerous than other contamination sources due to its cumulative impact over time through plant irrigation. Lead (Pb) is one of the most prevalent and hazardous heavy metals that significantly impede plant growth and development in terrestrial ecosystems. α- lipoic acid (ALA) is a naturally occurring dithiol antioxidant, strong ROS scavenger and metal chelator. Herein, this study was conducted to explore the role of exogenous ALA (0.1 mM) in reducing the Pb-phytotoxicity in tomato seedlings irrigated with Pb contaminated water (250 ppm for 45 days after transplanting). Exposing plants to Pb stress significantly inhibited plant growth, photosynthetic pigments, mineral homeostasis and cell membrane integrity compared to the control plants. In contrast, ALA application markedly revealed a significant improvement in these traits by reinforcing the antioxidant defense systems including superoxide dismutase, guaiacol peroxidase, catalase, glutathione reductase and the major reduced components of ascorbate glutathione cycle. Moreover, ALA significantly enhanced N, P, K, Ca and Fe, reduced Pb uptake and restricted the Pb-induced oxidative damage by reducing the hydrogen peroxide, malondialdehyde and inhibiting the activity of Lipoxygenase. The regression analysis exhibited that ALA demonstrated various significant relationships between the uptake of Pb and the major components of ascorbate glutathione cycle in both leaf and root. In conclusion, our findings deciphered the potential functions of ALA in alleviating Pb-phytotoxicity and enhancing the redox balance of tomato seedlings by enhancing the ratio between the reduced glutathione/oxidized glutathione and reduced ascorbate/dehydroascorbate.

## 1 Introduction

Globally, 14 to 17% of cropland is affected by toxic metal contamination, with estimates indicating that between 0.9 and 1.4 billion individuals reside in areas facing increased public health and ecological hazards (Hou et al., 2025). Lead (Pb) is a hazardous heavy metal readily absorbed by plant roots (Naziębło et al., 2025). It can accumulate in the soil due to industrial and human activities in many regions worldwide, leading to adverse effects on the food chain and human health (Kumar et al., 2020; Angon et al., 2024). Additionally, Pb can be deposited in the soil from atmosphere in polluted areas (Sun et al., 2006). In Saudi Arabia, elevated levels of Pb have been detected in soils near industrial zones and agricultural lands irrigated with untreated wastewater, raising concerns about food chain contamination and long-term ecological impacts (Al-Hammad and Abd El-Salam, 2016; Alturiqi et al., 2020). Lead metal ions can harm DNA and nuclear proteins, potentially causing cancer, cardiovascular diseases and Alzheimer’s disease (Huang et al., 2024; Shrivastav and Singh, 2024). In plants, although lead is a non-essential element, its presence can severely deteriorate the photosynthetic apparatus, respiration, nutrient uptake and redox balance (Lösch and Köhl et al., 1999; Sharma and Dubey, 2005; Kumar and Prasad, 2018). Elevated Pb concentrations promote the excessive generation of reactive oxygen species (ROS), which induce oxidative stress and severe damage to several plant species, including Indian senna (Qureshi et al., 2007), rice (Thakur et al., 2017), wheat (Navabpour et al., 2020) and tomato (Amubieya et al., 2024).

Generally, plants have evolved various effective mechanisms to cope with oxidative damage caused by heavy metals. Among these strategies, modulating the major components of ascorbate glutathione (AsA-GSH) cycle has received significant attention in previous reports (Liu et al., 2007; Jahan et al., 2020; Singh et al., 2021; Bashir et al., 2022). During this cycle, hydrogen peroxide (H_2_O_2_) is converted to water by ascorbate peroxidase (APX) in the presence of reduced ascorbate (AsA). The resultant dehydroascorbate (DHA) can be converted back to AsA using reduced glutathione (GSH) as an electron donor, leading to the formation of oxidized glutathione (GSSG). Subsequently, GSH can be regenerated from the reduction of GSSG by glutathione reductase (GR) in the presence of NADPH (Liu et al., 2007). Correspondingly, maintaining a high ratio between AsA/DHA and GSH/GSSG is crucial for adjusting the redox balance and scavenging ROS under stressful conditions (Gill et al., 2013).

Furthermore, plants develop several non-enzymatic and enzymatic antioxidants that orchestrate the network of redox signaling and coordinate various vital processes under stress conditions. In this regard, superoxide dismutase (SOD) has been found to protect plants from superoxide anions (O_2_·^−^) by converting them into H_2_O_2_ and O_2_ under heavy metal stress (Thakur et al., 2017; Jahan et al., 2020; Paul et al., 2024). This elimination of superoxide anions can be followed by activating catalase (CAT), which converts hydrogen peroxide (H_2_O_2_) into water (H_2_O) and oxygen (O_2_) (Stephenie et al., 2020). Guaiacol peroxidase (GPX) is also involved in H_2_O_2_ elimination and catalyzes the oxidation of many phenolic compounds under heavy metal stress conditions (Parmar et al., 2002). This response is highly important in lignin biosynthesis, which forms a physical barrier in the plant cell wall against the harmful effects of heavy metals (Michalak, 2006).

Alpha lipoic acid (ALA) is a lipophilic vitamin-like potent antioxidants with several benefits in clinical medicine (Estabragh et al., 2021). In animals, ALA is an important metabolic antioxidant capable of recycle other antioxidants such as glutathione (Busse et al., 1992), and vitamin E (Podda et al., 1994). It’s distinctive structure (a disulfide bond in the thiolane ring) and low molecular mass confer solubility in both water and lipids, making it an effective antioxidant in both its reduced and oxidized forms (Navari-Izzo et al., 2002). Recently, it has been used exogenously to improve plant tolerance to a wide array of abiotic stresses, including drought, salinity, alkalinity, heavy metals and heat stress (Ramadan et al., 2022; Yadav et al., 2022; Lee et al., 2023; Daler and Kaya, 2024). Exogenous ALA has been found to attenuate Pb phytotoxicity in wheat seedlings by affecting its uptake, accumulation, and transportation within root tissues and improving ROS detoxification and plant antioxidant capacity (Turk et al., 2018). Furthermore, Yadav et al. (2022) found that exogenous ALA mitigated Cd toxicity in tomato plants by regulating photosynthetic pigments, antioxidant enzymes, and nitrogen-assimilating related enzymes. Therefore, ALA has been suggested as an optimal and promising antioxidant, metal chelator, ROS scavenger and organizer for other antioxidants under stress conditions (Navari-Izzo et al., 2002).

Irrigation with Pb-contaminated water poses a significant threat to agricultural sustainability in many regions worldwide due to its harmful cumulative impacts on soil and plants over time. Up to now, no scientific report has been found regarding how ALA can mediate the tolerance of tomato plants to Pb toxicity specifically, which is caused by Pb-contaminated water. Therefore, this study was conducted to investigate: (1) the role of ALA in regulating root/shoot growth ratio, photosynthetic pigments and nutrient homeostasis; (2) the role of ALA in controlling lipoxygenase activity and restoring cell membrane integrity and water status; (3) the role of ALA in regulating the non-enzymatic antioxidant defense systems, specifically the major components of ascorbate–glutathione cycle, including reduced glutathione, ascorbate, oxidized glutathione and dehydroascorbate; (4) the role of ALA in orchestrating the activities and functions of antioxidant enzymes including superoxide dismutase, catalase, peroxidase, ascorbate peroxidase, glutathione reductase and dehydroascorbate reductase; and (5) to understand the relationship between the concentration of Pb and modifying the major components of ascorbate–glutathione cycle to achieve the balance of plant redox status. To our knowledge, this is the first report to explore the mechanisms involved in the ALA-mediated Pb-stress tolerance in tomato seedlings irrigated with Pb-contaminated water.

## 2 Materials and methods

### 2.1 Plant material and growth conditions

Tomato (*Solanum lycopersicum* L. Cv. Super Strain B) seeds were sterilized with 0.5% (v/v) sodium hypochlorite solution for 10 min, followed by rinsing four times with distilled water for 15 min. The sterilized seeds were sown in trays containing 50 individual cells (4 cm × 4 cm × 6 cm) filled with pre-washed sand and irrigated daily with ¼ strength Hoagland solution. The trays were then placed under greenhouse conditions for one month at 28°C ± 3, 16 h light and 8 h dark cycle. Seedlings with a similar morphology (displaying four true leaves) were transplanted into pots containing 4 kg of pre-washed sand, with one seedling per pot. The optimal level of Pb toxicity (250 ppm) was determined from a range of concentration (0, 25, 50, 100, 250, 500, and 1000 ppm) of Pb(NO_3_)_2_. This determination was made using an initial experiment based on plant fresh weight and the C_50_ value (the concentration at which growth is reduced by 50% compared to non-stressed control plants). In this preliminary experiment, seedlings were grown for an additional 36 days under greenhouse conditions and irrigated regularly and mutually with 350 mL ½ strength Hogland solution in the first day and in the second day with distilled water containing different concentrations of Pb(NO_3_)_2_.

### 2.2 Pb and ALA treatments

The experiment included four treatments: (1) control, (2) ALA, (3) Pb, and (4) Pb+ALA. Each pot with a single seedling was irrigated day by day with 350 mL of ½ strength Hoagland solution and 350 mL of distilled water, respectively. To apply Pb stress, the distilled water was contaminated with Pb(NO_3_)_2_ at 250 ppm. ALA was applied as a foliar application at 0.1 mM every three days. This concentration was selected according to a previous study (Yadav et al., 2022). Tween 20 at 0.05% was used as a non-surfactant with all foliar applications. ALA-untreated plants were sprayed with distilled water+Tween 20. The trial was laid out as a completely randomized design (CRD) with 5 replicates. The rationale for alternating irrigation with distilled water and nutrient solution was to control the increase in soil salinity, a technique known as leaching. Additionally, adding the Pb(NO_3_)_2_ solution separately prevented any modification in the nutrient solution for all treatments and allowed for the study of its cumulative impact on tomato seedlings over time.

### 2.3 Quantification of leaf pigments

Leaf pigments were quantified following Lichtenthaler (1987). Briefly, 0.5 g of plant tissue was extracted in 10 mL of 80% acetone and centrifuged at 10,000 rpm. The absorbance of the supernatant was measured at 645, 663, and 470 nm to determine chlorophyll a, chlorophyll b, and carotenoids, respectively according to the following equations:


$$\text{Chl}\;a(\text{mg}.{\text{ g}}^{-1}\;\text{FW})=(12.25\times \text{A}663-2.79\times \text{A}645)\times 0.02$$

$$\text{Chl}\;b(\text{mg}.{\;\text{ g}}^{-1}\;\text{FW})=(21.50\times \text{A}645-5.1\times \text{A}663)\times 0.02$$

$$\text{Car}(\text{mg}.{\text{ g}}^{-1}\;\text{FW})=((1000\times \text{A}470-1.8\times \text{Chl}\;a-85.02\times \text{Chl}\;b)/198)\times 0.02$$

### 2.4 Quantification of leaf relative water content and electrolyte leakage

Leaf relative water content (RWC) was calculated according to Weatherley (1950) using the formula:


$$RWC\;(\%)=\frac{FW-DW}{TW-DW}\times 100$$

Where FW = fresh weight, TW = turgid weight, and DW = dry weight.

Electrolyte leakage (EL) was measured to assess cell membrane stability according to Prado et al. (2015) with some modification using the formula:


$$EL\;(\%)=\frac{EL1}{EL2}\times 100$$

Where EL1= The initial conductivity after 1 hour deionized water at 28°C, and EL2 = final conductivity after heating at 95°C for 20 min.

### 2.5 Quantification of nutrients

Leaf and root samples were oven-dried at 68°C for 48 hours. Dried samples (0.5 g) were ground and digested using sulfuric acid and hydrogen peroxide. Nitrogen (N) was determined using Kjeldahl method (Bradstreet, 1954); phosphorus (P) by the vanadomolibdophosphoric acid colorimetric method (Kuo, 1996); potassium (K) by flame spectrophotometry; and calcium (Ca), iron (Fe) and lead (Pb) by atomic absorption spectrophotometry according to Cottenie et al. (1982).

### 2.6 Quantification of lipid peroxidation, hydrogen peroxide and lipoxygenase activity

Lipid peroxidation was assessed by measuring malondialdehyde (MDA) content, following Heath and Packer (1965) with some modifications. Fresh plant tissue (0.5 g) was homogenized in 5 mL of 5% trichloroacetic acid (TCA) and centrifuged at 4000 rpm for 10 min at 4°C. The supernatant (2 mL) was mixed with 2 mL of 5% TCA containing 0.67% (m/v) thiobarbituric acid, heated at 95°C for 30 min, and rapidly cooled. Absorbance was measured at 532 nm and corrected for non-specific turbidity at 600 nm. Hydrogen peroxide (H_2_O_2_) concentration was determined according to Velikova et al. (2000), modified by Ibrahim et al. (2020). Plant tissue (0.5 g) was homogenized in 3 mL TCA and centrifuged at 10,000 rpm at 4°C for 10 min. The supernatant (0.75 mL) was mixed with 0.75 mL of 10 mM potassium phosphate buffer (pH 7) and 1.5 mL of 1 M KI. Absorbance was measured at 390 nm. Lipoxygenase (LOX) activity was measured following Axelrod et al. (1981) with some modifications. The reaction was initiated by adding 250 µl of crude extract to 500 µl of 0.4 mM linoleic acid and 2.25 mL of 50mM potassium phosphate buffer (pH 6.5). The increase in absorbance was measured at 234 nm.

### 2.7 Quantification of ascorbate-glutathione cycle components

Plant tissue (0.5 g) was frozen in liquid nitrogen and homogenized in 5 mL of 10% TCA containing 1.5 mM EDTA. The homogenate was centrifuged for 15 min at 12000 rpm. The supernatant was neutralized with 5% sodium phosphate buffer (pH 7.5) at a ratio of 1:50. Total glutathione was quantified using 5,5′-dithio-bis (2-nitrobenzoic acid) (DTNB) in the presence of glutathione reductase, as described by Griffith (1980) and modified by Ding et al. (2009). Absorbance was measured at 412 nm. Oxidized glutathione (GSSG) was determined by incubating the extract with 2-vinylpyridine to remove GSH, followed by the same assay. Reduced glutathione (GSH) was calculated as the difference between total glutathione and GSSG.

Ascorbate (AsA), dehydroascorbate (DHA), and total ascorbate were measured following Gillespie and Ainsworth (2007) with some modification. Plant tissue (0.4 g) was homogenized in 3 mL of 6% TCA and centrifuged for 10 min at 10000 rpm and 4°C. Total ascorbate was measured by mixing the extract with potassium phosphate buffer, water, TCA, H_3_PO_4_, α-α’-bipyridyl, and FeCl_3_, incubating at 37°C for 1 hour, and measuring absorbance at 525 nm. Reduced ascorbate (AsA) was measured similarly, but with the addition of dithiothreitol (DTT) and 0.5% N-ethylmaleimide (NEM) to remove the excess of DTT. Absorbance was observed at 525 nm. DHA was calculated as the difference between the total ascorbate and AsA.

### 2.8 Determination of antioxidant enzyme activities

Fresh plant tissue (0.5 g) was homogenized in 4 mL of 0.1M potassium phosphate buffer (pH 7.0) supplemented with 1% (w/v) polyvinylpyrrolidone (PVP) and 0.1 mM EDTA. The homogenate was centrifuged at 10,000 rpm for 15 minutes, and the supernatant was used as the enzyme extract. Protein content was determined using the Bradford (1976). Glutathione reductase (GR) activity was measured by monitoring NADPH oxidation at 340 nm (Foyer et al., 1995). Dehydroascorbate reductase (DHAR) activity was measured by measuring ascorbic acid production at 265 nm (Nakano and Asada, 1981). Superoxide dismutase (SOD) activity was determined by its ability to inhibit NBT reduction (Giannopolitis and Ries, 1977). Catalase (CAT) activity was measured by H_2_O_2_ decomposition at 240 nm (Patra et al., 1978). Ascorbate peroxidase (APX) activity was determined by ascorbic acid oxidation at 290 nm Nakano and Asada (1981). Peroxidase (POD) activity was measured using guaiacol and H_2_O_2_ (Rao et al., 1996).

### 2.9 Statistical analysis

Data were analyzed using one-way ANOVA in SAS^®^ 9.1. 3 software, followed by Duncan’s multiple range test to determine significant differences at P<0.05. Results are presented as means of five replicates ± standard error (SE).

## 3 Results

### 3.1 Determination of Pb toxicity and improving plant growth using α lipoic acid

**Figure 1:**
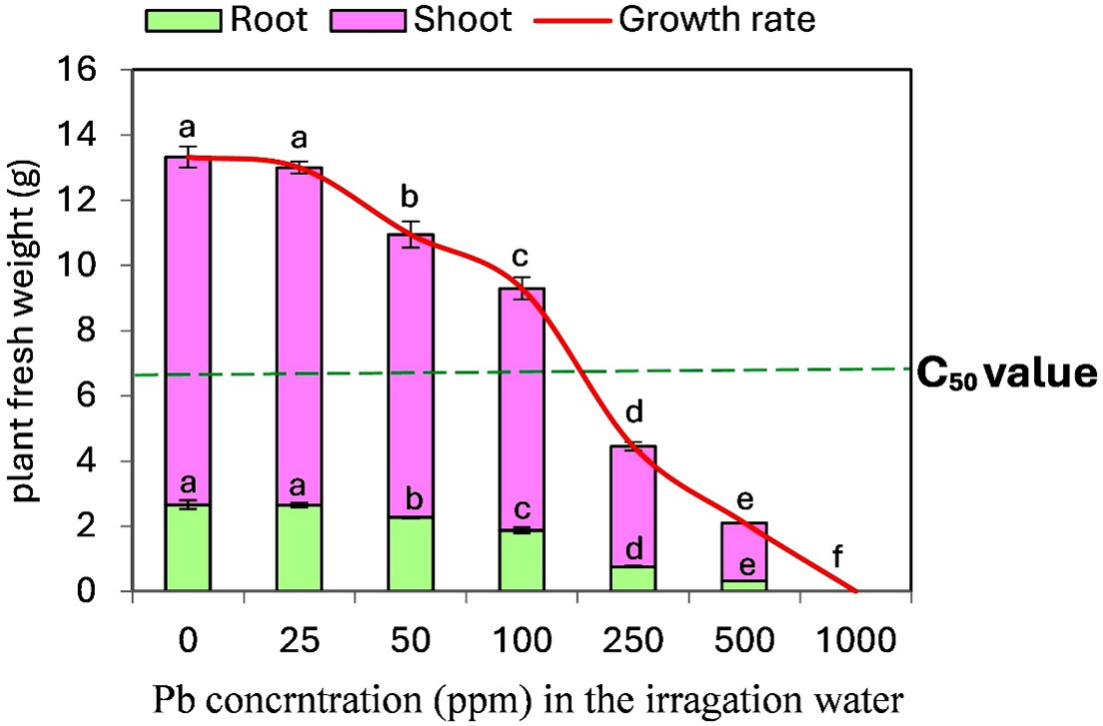
Development and growth responses of tomato seedlings to different concentrations of Pb (ppm). The dotted green line indicates C_50_ value (50 % of non- stressed plants). Data presented in each column are means of 5 replicates ± SE according to Duncan's multiple range test at P<0.05.

To determine the Pb-associate C_50_ value in tomato seedlings, we optimized an experimental setting as illustrated in **Figure 1**. An increase in Pb concentration from 0 ppm (control) to 1000 ppm resulted in a progressive decline in both shoot and root system growth. This reduction fell below the C_50_ value (50% of non-stressed plants) when seedlings were treated with Pb concentrations exceeding 100 ppm in soil. Consequently, a concentration of 250 ppm was deemed optimal for studying the effect of ALA in mitigating the harmful effects of Pb on tomato plants at toxic levels under the conditions of this study. Conversely, a Pb concentration of 1000 ppm completely inhibited plant growth.

**Figure 2:**
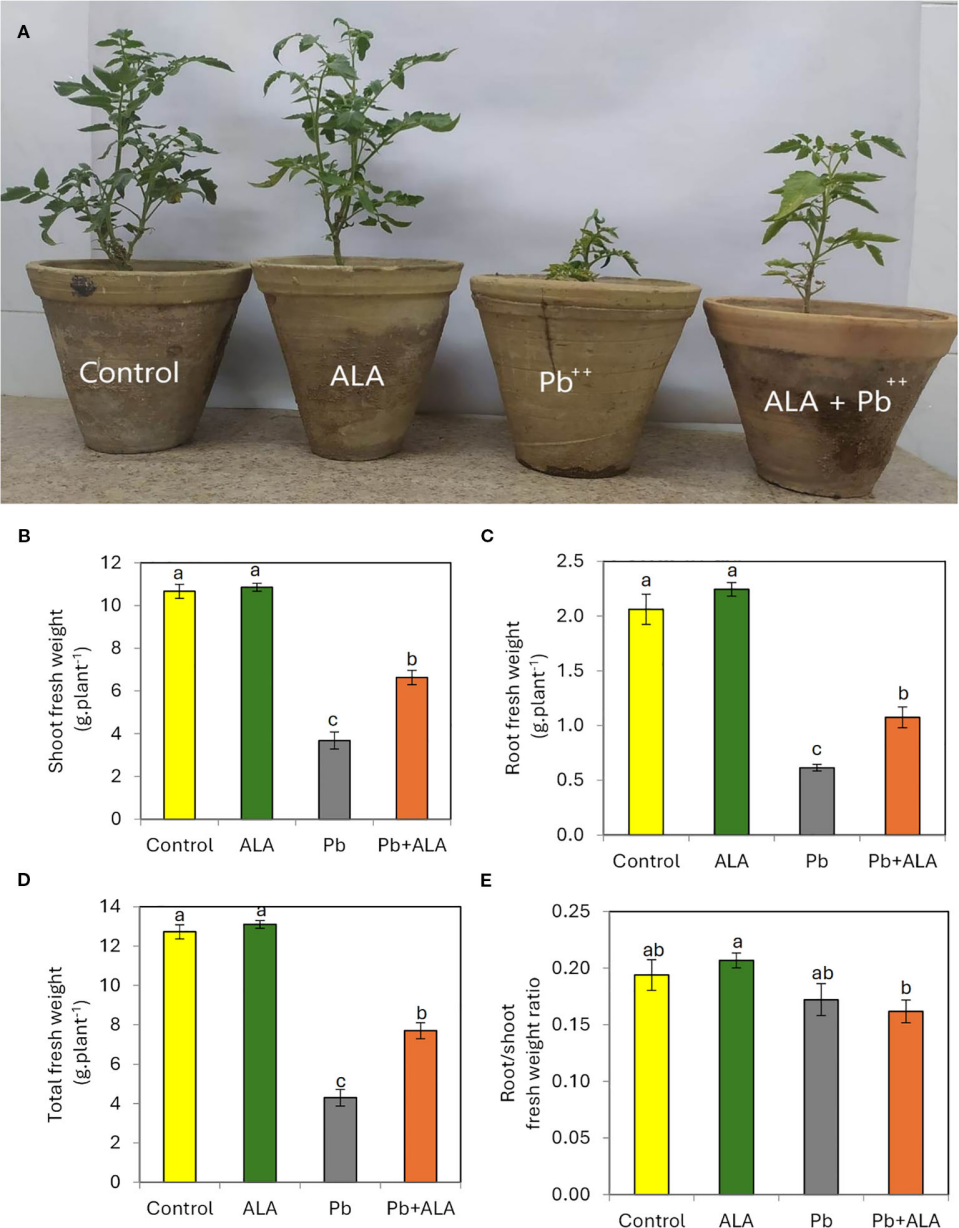
Effect of ALA foliar spraying at 0.1 mM on plant growth of tomato seedlings grown under non-stress and Pb toxicity (250 ppm). **(A)** growth and development of tomato plants, **(B)** shoot fresh weight, **(C)** Root fresh weight, **(D)** total plant fresh weight and **(E)** root/shoot fresh weight ratio. Data presented in each column are means of 5 replicates ± SE according to Duncan's multiple range test at P≤0.05.

Plants treated with Pb exhibited distinct leaf chlorosis symptoms and a significant reduction in plant growth compared to unstressed plants (**Figures 2A–E**). Specifically, plants exposed to Pb alone revealed a significant decrease in shoot fresh weight (-65.5%), root fresh weight (-70.2%), and total plant fresh weight (-66.2%) compared to the control seedlings. Conversely, plants treated with ALA demonstrated a positive recovery from the harmful effects of Pb toxicity on their growth parameters. The root/shoot fresh weight ratio showed a slight decrease in ALA-treated plants compared to the control, suggesting that ALA relatively improved the growth of root system, which is directly impacted by the toxic Pb ions in the soil. However, no significant changes were observed between ALA-treated and non-treated plants in the absence of Pb stress.

### 3.2 α lipoic acid enhances leaf pigments, relative water content and controls electrolyte leakage under Pb stress

**Table 1** illustrates the effect of Pb stress on plant physiology. Plants exposed to Pb exhibited a significant reduction in Chl a (-47.3%), Chl b (-37.5%), total Chl (-43.9%), carotenoids (-40.3%) and RWC (-23.3%), while EL increased by 82.1% compared to the control. In contrast, plants treated with Pb+ALA demonstrated significant improvement in this respect. Additionally, ALA treatment under non-stress conditions resulted in a significant increase in the total Chl (13.9%), primarily due to a significant enhancement of Chl b content compared to the control seedlings. These results imply that ALA, acting as an effective antioxidant, can protect the photosynthetic apparatus from Pb stress by modulating the Chl a/b ratio, maintaining leaf water status, and preserving cell membrane integrity.

**Table 1:** Effect of ALA (0.1 mM) on leaf pigment concentration, relative water content (RWC) and electrolyte leakage in tomato seedlings exposed to Pb toxicity through irrigation with 250 ppm Pb(NO_3_)_2_-contaminated water.

Treatments	Chl a (mg. g^-1^ FW)	Chl b (mg. g^-1^ FW)	Total Chl (mg. g^-1^ FW)	Carotenoids (mg. g^-1^ FW)	RWC (%)	Electrolyte leakage (%)
Control	1.53 ± 0.09 a	0.77 ± 0.03 b	2.30 ± 0.10 b	0.55 ± 0.022 a	89.5 ± 1.59 a	29.86 ± 2.22 c
ALA	1.68 ± 0.11 a	0.94 ± 0.02 a	2.62 ± 0.11 a	0.52 ± 0.032 a	91.2 ± 1.61 a	26.30 ± 1.91 c
Pb	0.80 ± 0.03 c	0.48 ± 0.04 d	1.29 ± 0.05 d	0.33 ± 0.008 c	68.7 ± 2.61 c	54.37 ± 0.89 a
Pb + ALA	1.07 ± 0.06 b	0.63 ± 0.02 c	1.70 ± 0.08 c	0.40 ± 0.017 b	80.8 ± 1.67 b	41.26 ± 1.01 b

Data presented in each column are means of 5 replicates ± SE according to Duncan’s multiple range test at P ≤0.05.

### 3.3 α lipoic acid reduces Pb absorption and enhances plant nutrient homeostasis under Pb stress

To elucidate the effect of ALA on the Pb uptake and nutrient homeostasis under Pb stress, the concentrations of N, P, K, Ca, Fe and Pb were determined (**Table 2**). Under Pb stress, Pb uptake increased approximately 1.62-fold in leaves and 3.97-fold in roots compared to the unstressed plants. In contrast, the concentrations of N, P, K and Fe in both leaves and roots, and Ca in roots, significantly decreased in the Pb-stressed plants relative to the controls. However, plants treated with ALA exhibited a significant increase in the concentrations of N, P, K and Fe in both roots and leaves. Conversely, Ca concentration exhibited a contrasting trend between the roots and leaves. In leaves, plants treated with Pb + ALA showed a decrease in Ca content compared to ALA-untreated plants under Pb stress. These results suggest that ALA plays a pivotal role in maintaining the nutrient homeostasis in tomato plants under Pb stress. Concurrently, ALA may facilitate the accumulation of substantial amounts of Ca in roots. This response may reinforce cell wall structure, thereby influencing Pb deposition and its detoxification away from the internal sensitive tissues.

**Table 2:** Effect of ALA (0.1 mM) on nutrient homeostasis and Pb uptake in leaves and roots of tomato plants exposed to Pb toxicity through irrigation with 250 ppm Pb(NO_3_)_2_-contaminated water.

Treatments	N (mg. g^-1^ DW)	P (mg. g^-1^ DW)	K (mg. g^-1^ DW)	Ca (mg. g^-1^ DW)	Fe (mg. kg^-1^ DW)	Pb (mg. kg^-1^ DW)
Leaf						
Control	76.1 ± 2.8 b	2.70± 0.12 b	32.2 ± 1.01 b	9.3 ± 0.54 b	246 ± 10.5 a	14.0 ± 0.99 bc
ALA	89.6 ± 1.6 a	3.52 ± 0.05 a	38.9 ± 1.63 a	10.9 ± 0.50 a	280 ± 22.2 a	10.7 ± 0.68 c
Pb	54.3 ± 2.9 d	1.49 ± 0.06 d	21.1 ± 1.56 d	8.7 ± 0.19 b	167 ± 16.7 b	22.7 ± 1.84 a
Pb + ALA	66.8 ± 2.0 c	2.12 ± 0.03 c	27.6 ± 0.30c	7.3 ± 0.15 c	233 ± 10.9 a	15.9 ± 1.14 b
Root						
Control	54.7 ± 0.63 b	2.28 ± 0.09 b	23.7 ± 1.12 b	9.8 ± 0.18 b	394 ± 20.5 a	20.6 ± 1.47 c
ALA	61.8 ± 2.4 a	2.84 ± 0.24 a	26.7 ± 0.41a	11.0 ± 0.08 a	359 ± 9.0 a	17.3 ± 1.09 c
Pb	41.5 ± 2.7 d	1.46 ± 0.12 c	13.6 ± 0.66 d	5.7 ± 0.23 d	220 ± 11.5 b	81.9 ± 3.1 a
Pb + ALA	48.0 ± 1.9 c	1.61 ± 0.06 c	18.8 ± 0.39 c	8.1 ± 0.18 c	378 ± 15.9 a	48.9 ± 5.0 b

Data presented in each column are means of 5 replicates ± SE according to Duncan’s multiple range test at P ≤0.05.

### 3.4 α lipoic acid reduces the oxidative damage and inhibits the lipoxygenase activity

**Figure 3:**
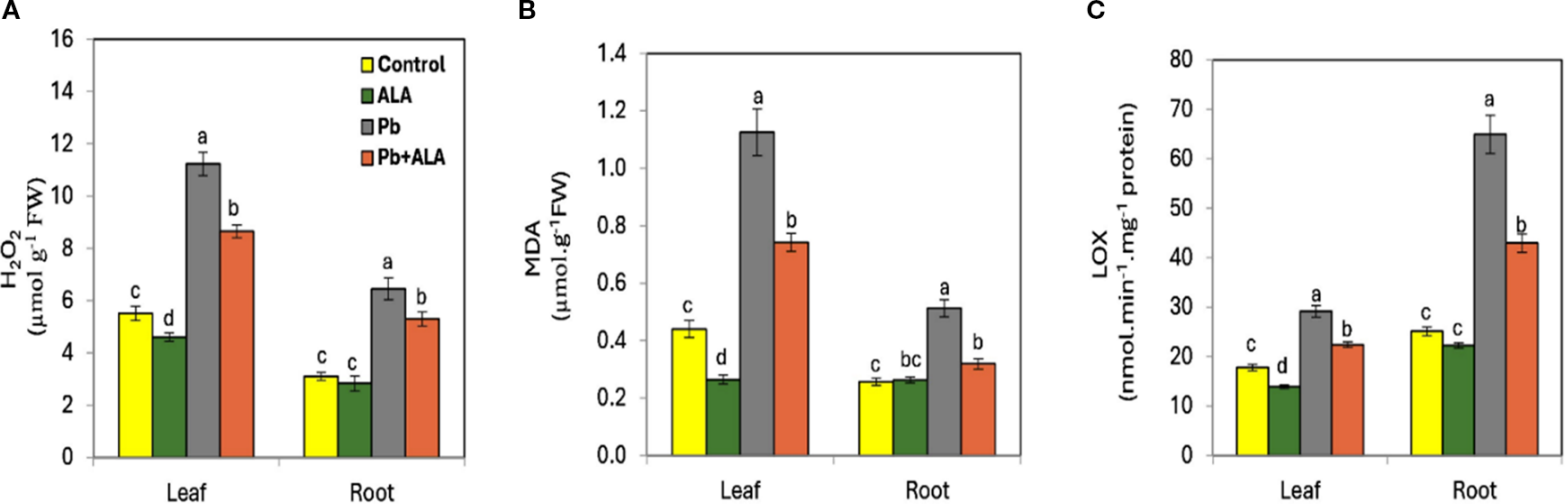
Effect of ALA foliar spraying at 0.1 mM on the concentration of hydrogen peroxidase; H_2_O_2_, malondialdehyde; MDA and lipoxygenase; LOX activity of tomato seedlings irrigated with contaminated water with 250 ppm Pb(NO_3_)_2_.. **(A)** H_2_O_2_, **(B)** MDA, **(C)** LOX. Data presented in each column are means of 5 replicates + SE according to Duncan's multiple range test at P≤0.05.

Under Pb stress, both leaves and roots exhibited significant increases in H_2_O_2_ and MDA concentrations compared to unstressed plants (**Figures 3A, B**). These increases were more pronounced in leaves than in roots. Conversely, LOX activity was higher in roots than in leaves under Pb stress (**Figure 3C**). Exogenous ALA significantly reduced H_2_O_2_, MDA and LOX activity in stressed plants compared to ALA-untreated plants under the same Pb stress conditions. These results suggest that ALA plays a crucial role in mitigating oxidative damage and lipid peroxidation under Pb stress. By reducing these factors, ALA likely decreases the availability of LOX substrates, thereby limiting the conversion of polyunsaturated fatty acids into hydroperoxy fatty acids. This response may be attributed to ALA’s potent antioxidant properties, which protect cell membrane structure and function under Pb stress.

### 3.5 α lipoic acid modulates the balance between the major components of ascorbate-glutathione cycle under Pb stress conditions

**Figure 4:**
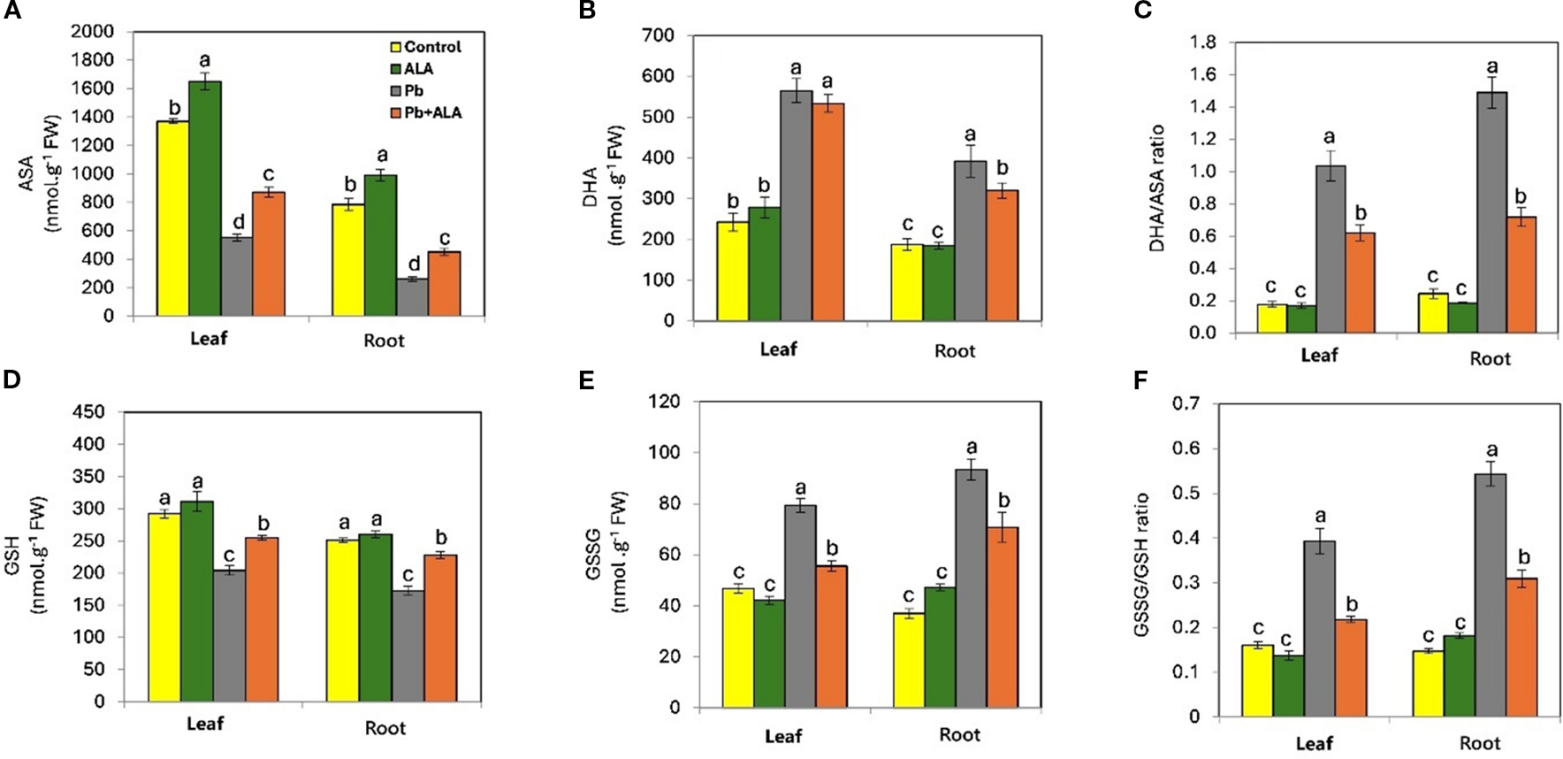
Effect of ALA foliar spraying at 0.1 mM on the concentration of major components of ASA-GSH cycle of tomato seedlings irrigated with contaminated water with 250 ppm Pb(NO_3_)_2._ **(A)** reduced ascorbate (ASA), **(B)** dehydroascorbate (DHA), **(C)** DHA/ASA ratio, **(D)** reduced glutathione (GSH), **(E)** oxidized glutathione (GSSG) and **(F)** GSSG/GSH ratio. Data presented in each column are means of 5 replicates ± SE according to Duncan's multiple range test at P < 0.05.

To investigate the effect of ALA on the cellular redox balance under Pb stress, we measured the major components of the ASA-GSH cycle (**Figures 4A–F**). Under non-stress conditions, the reduced redox status was dominant in both leaves and roots, as evidenced by higher levels of ASA and GSH compared to their oxidized forms, DHA and GSSG respectively. This was further supported by the low DHA/ASA (0.178 and 0.244) and GSSG/GSH (0.161 and 0.147) ratios in leaves and roots respectively. These results indicate that tomato seedlings maintained approximately 80-90% of their reduced cellular redox status under non-stress conditions. In contrast, Pb-stressed plants showed significant increases in DHA (132.8%, 108.7%) and GSSG (69.6%, 152.4%) in leaves and roots, respectively, compared to the control plants. This shift in redox balance was reflected by a 5.8-fold and 6.1-fold increase in the DHA/ASA ratio, and a 2.4-fold and 3.7-fold increase in the GSSG/GSH ratio, in leaves and roots respectively, compared to unstressed plants. However, Pb-stressed plants treated with ALA exhibited a significant improvement in their redox balance, characterized by increased ASA (23.2%, 24.1%) and GSH (17.4%, 22.1%) levels in leaves and roots respectively, compared to Pb-stressed plants without ALA treatment. Consequently, ALA treatment effectively restored the redox balance, as evidenced by significantly decreased DHA/ASA and GSSG/GSH ratios in leaves and roots of Pb-stressed plants.

### 3.6 α Lipoic acid modulates antioxidant enzyme activities under normal and Pb stress conditions

**Figure 5:**
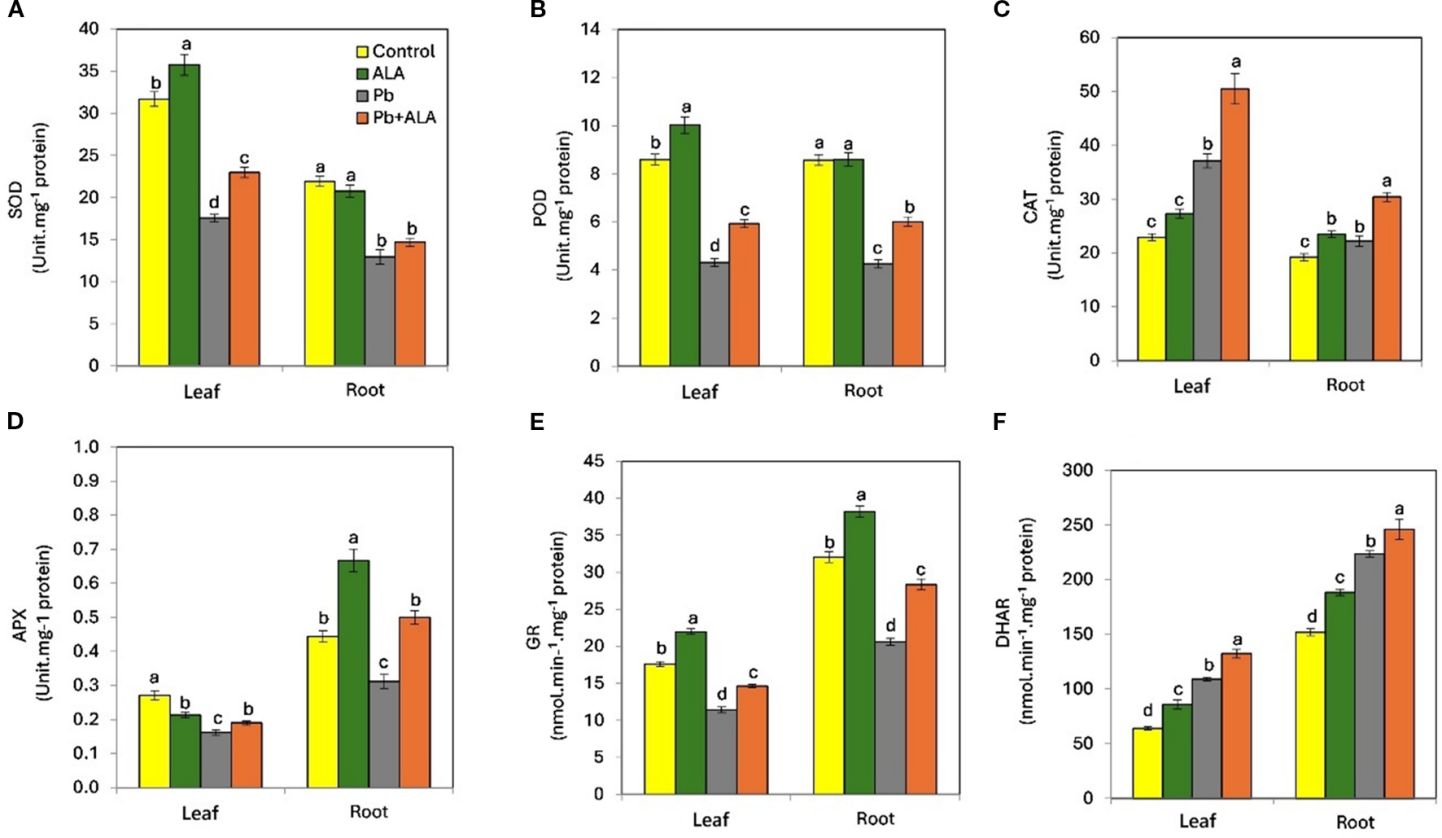
Effect of ALA foliar spraying at 0.1 mM on the activities of antioxidant enzymes of tomato seedlings irrigated with contaminated water with 250 ppm Pb(NO_3_)_2_. **(A)** superoxide dismutase (SOD), **(B)** peroxidase (POD), **(C)** catalase (CAT), **(D)** ascorbate peroxidase (APX), **(E)** glutathione reductase (GR) and **(F)** dehydro ascorbate reductase (DHAR). Data presented in each column are means of 5 replicates ± SE according to Duncan's multiple range test at P ≤ 0.05.

To further investigate the effect of ALA in mitigating the detrimental effects of Pb stress and regulating antioxidant defense systems in tomato seedlings, we examined the activities of antioxidant enzymes, including SOD, POD, CAT, APX, GR and DHAR (**Figures 5A–F**). Plants exposed to Pb stress exhibited a significant decrease in the activities of SOD (-44.6%, -40.9%), POD (-49.7%, -50.3%), APX (-40.2%, -29.8%) and GR (-35.2%, -31.6%) in both leaves and roots, compared to control plants. Conversely, CAT (61.8%, 15.4%) and DHAR (48.4%, 57.4%) activities increased in the leaves and roots respectively, under Pb stress compared to the unstressed plants. However, plants treated with ALA under Pb stress conditions showed a significant increase in the activities of SOD (30.8%, 13.4%), POD (37.4%, 41.1%), CAT (33.2%, 36.4%), APX (17.5%, 60.4%), GR (28.2%, 37.4%) and DHAR (23.6%, 19.8%) in both leaves and roots, compared to Pb-stressed plants without ALA. These results suggest that ALA can effectively alleviate Pb-induced oxidative damage in tomato seedlings by modulating the activities of various antioxidant enzymes.

### 3.7 α lipoic acid affects the relationship between the components of ascorbate-glutathione cycle and Pb concentration

**Figure 6:**
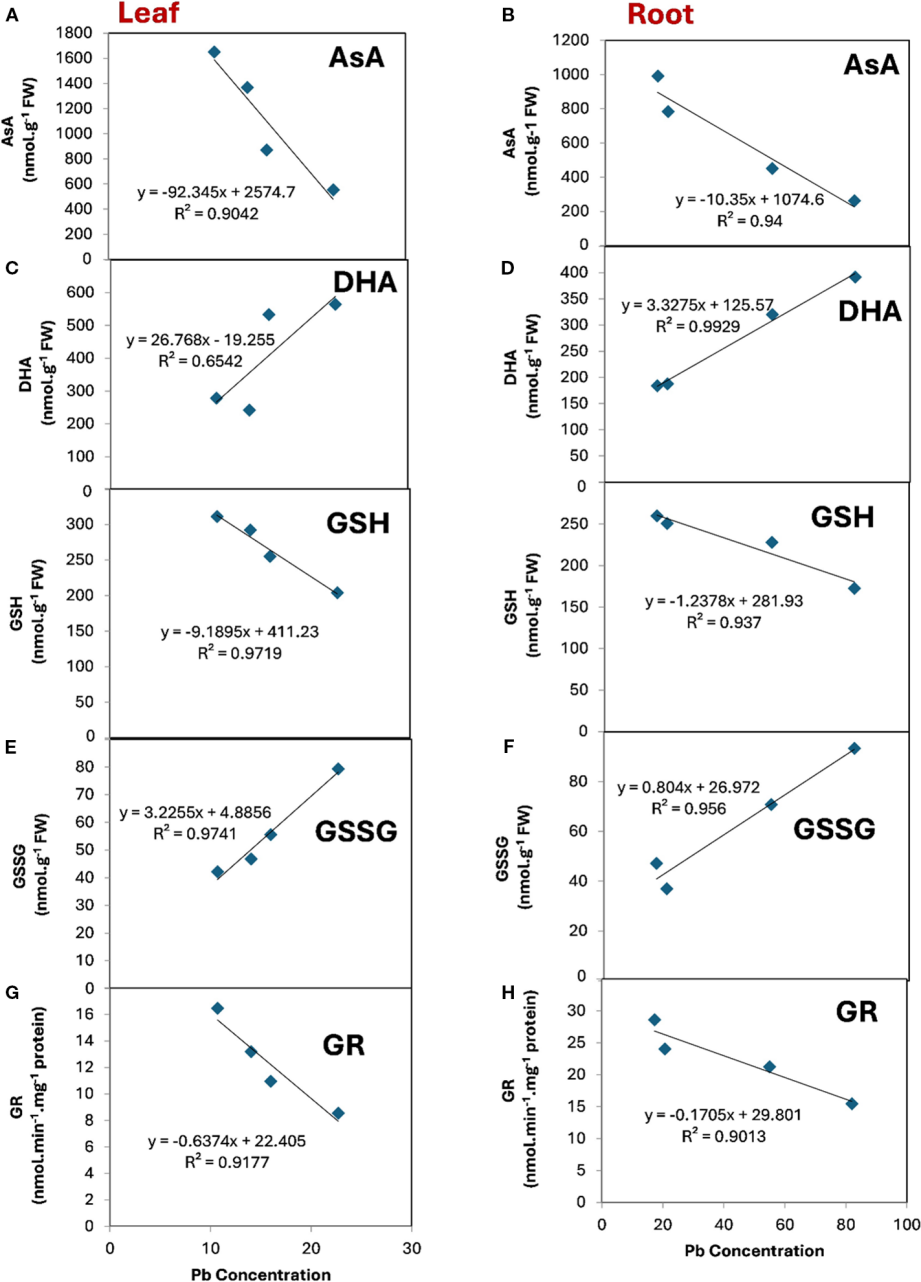
The relationship between the major components of ascorbate-glutathione cycle and Pb concentration under the treatment of ALA in both leaf (left) and root (right) tissue. **(A, B)**, ascorbate (ASA), **(C, D)**, dehydroascorbate (DHA), **(E, F)**, reduced glutathione (GSH), **(G, H)**, oxidized glutathione (GSSG), **(I, J)**, glutathione reductase (GR) in both leaves and roots respectively.

α Lipoic acid can modulate the relationship between the major components of ascorbate-glutathione cycle and the uptake of Pb by leaf and root (**Figures 6A–J**). It can be observed that AsA, GSH and GR were negatively and significantly correlated with the concentration of Pb in both leaf and root tissue. Conversely, DHA and GSSG revealed a positive correlation with the concentration of Pb in both leaf and root tissue. However, this relationship was not significant between DHA and Pb concentration (**Figure 6C**) in the leaf tissue where R^2^ was 0.6542. The highest significant and positive correlation was observed between DHA and Pb concentration in the root tissue (**Figure 6D**) where the R^2^ was 0.9929. In contrast the highest significant and negative correlation was noticeable between GSH and Pb concentration in the leaf tissue where the R^2^ was 0.9719.

**Figure 7:**
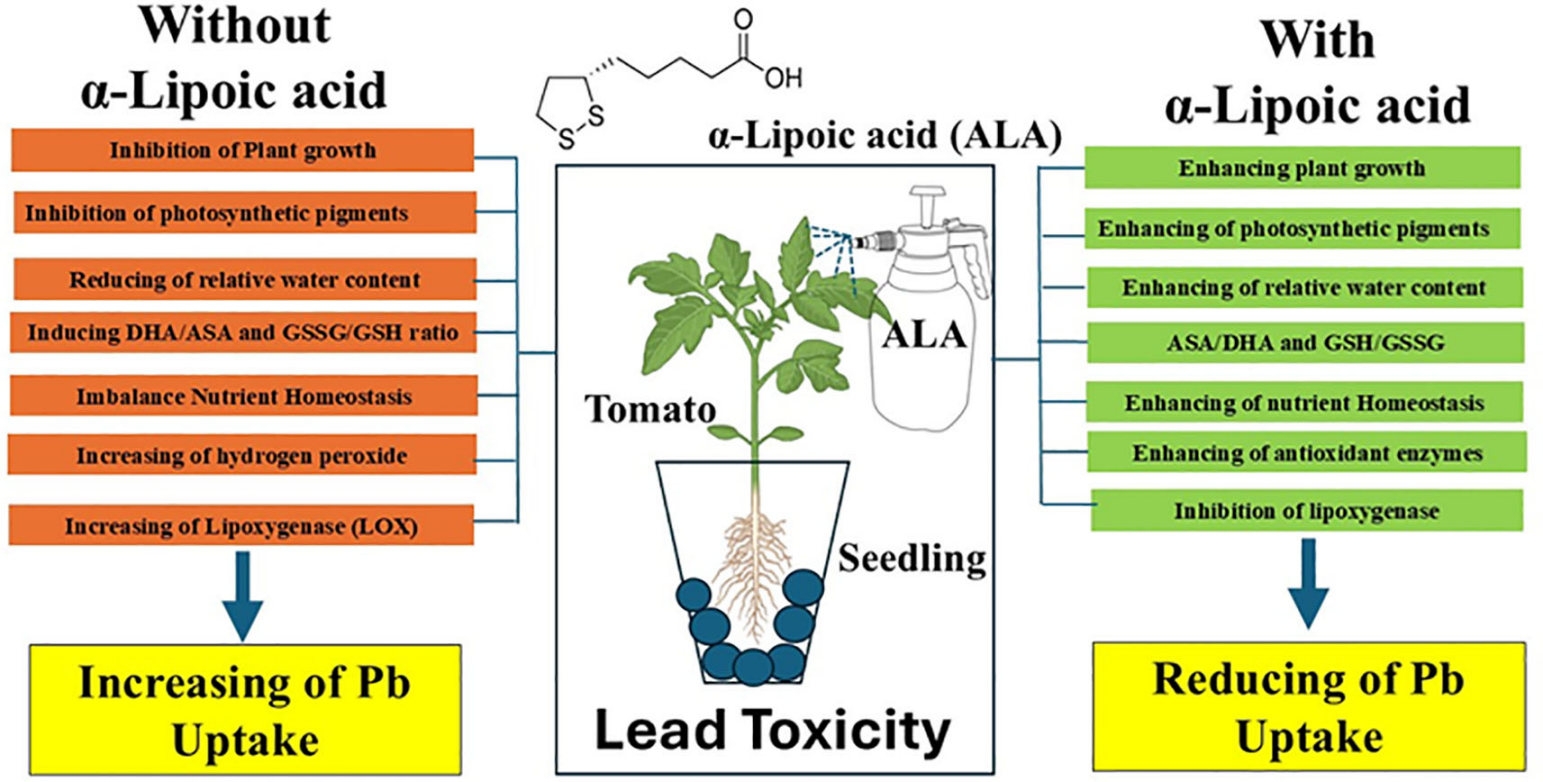
A simplified model for the role of exogenes ALA in mitigating Pb toxicity in tomato seedlings.

## 4 Discussion

Heavy metal contamination significantly impedes agricultural productivity worldwide. The germination and initial growth stages of seedlings are among the most vulnerable phases of plant life cycle to heavy metals (Li et al., 2005; Seneviratne et al., 2019). Lead (Pb), a non-essential metal, has extremely harmful effects on plant growth and development (Qureshi et al., 2007; Kumar and Prasad, 2018; Amubieya et al., 2024). In this study, plants exposed to Pb exhibited a significant decline in growth parameters compared to unstressed plants. These findings align with previous research on various plant species, including tomato (Akinci et al., 2010), rice (Awan et al., 2015) and sunflower (Saleem et al., 2018). Pb disrupts microtubules organization in the root meristem of maize seedlings, which is essential for cell division and proper plant cell cycle function (Eun et al., 2000). Additionally, Pb negatively affects nutrient homeostasis and key enzymes involved in photosynthesis and respiration, which are critical for CO_2_ fixation and ATP production (Parys et al., 1998; Dalyan et al., 2020).

In contrast, plants treated with ALA showed significant improvement in growth parameters under Pb stress. Exogenous ALA has emerged as a promising strategy to mitigate various abiotic stresses, including drought (Sezgin et al., 2019), salinity (Youssef et al., 2021), alkalinity (Ramadan et al., 2022), heat stress (Lee et al., 2023) and heavy metals (Turk et al., 2018; Yadav et al., 2022). These effects are closely linked to ALA’s antioxidant properties, which effectively detoxify ROS under stress conditions (Terzi et al., 2018; Elkelish et al., 2021; Behairy et al., 2024). However, a slight decrease in the root/shoot ratio was observed between ALA-treated and non-treated plants under Pb stress (**Figure 2E**), suggesting that ALA is more effective on the shoot system than on roots. This may be due to the root system’s direct contact with Pb in the soil, making it more susceptible to Pb accumulation compared to the shoot system (Dalyan et al., 2020).

Pb, a non-biodegradable and toxic metal, disrupts chlorophyll biosynthesis, inhibits photosynthesis, reduces leaf water status and damages cell membranes in various plant species (Parys et al., 1998; Awan et al., 2015; Leal-Alvarado et al., 2016). These effects are attributed to the destruction of cell membranes and ultrastructure of chloroplasts under Pb stress (Zhou et al., 2017). Pb also increases chlorophyllase (*Chlase*) activity, leading to chlorophyll degradation (Salavati et al., 2021). Furthermore, Pb negatively affects the content of δ-aminolevulinic acid and the activity of δ-aminolevulinic acid dehydrase, both of which are essential for chlorophyll biosynthesis in higher plants (Cenkci et al., 2010). This disruption in the photosynthetic apparatus can lead to a significant reduction in the components of plant’s carbon skeleton and isoprenoid substrates required for carotenoid biosynthesis (Cazzonelli and Pogson, 2010). In this study, ALA application under Pb stress significantly improved Chl a, Chl b, total Chl, carotenoids and RWC, while reducing EL compared to non-treated plants (**Table 1**). Previous studies on maize seedlings under drought stress have shown that ALA enhances chlorophyll biosynthesis by upregulating the expression of the magnesium chelatase (*Mg-CHLI*) gene, a key chlorophyll biosynthetic genes, while downregulating chlorophyllase (*Chlase*) to prevent chlorophyll degradation (Sezgin et al., 2019). Evidence suggests that ALA enhances RWC by improving cell membrane integrity through its antioxidant properties and ROS scavenging under stress conditions (Elkelish et al., 2021; Youssef et al., 2021; Ramadan et al., 2022).

Lead toxicity can hinder nutrient uptake and disrupt ion homeostasis in plants (Akinci et al., 2010). Several studies have demonstrated that exogenous ALA can improve ion homeostasis in plants under normal (Türk, 2023) or abiotic stress conditions (Gorcek and Erdal, 2015; Youssef et al., 2021; Ramadan et al., 2022). However, the mechanism by which ALA facilitates nutrient uptake and transport remains unclear. In this study, ALA significantly increased the root and leaf content of N, P, K, Fe under Pb toxicity. In contrast, Ca exhibited a contrasting trend between roots and leaves in response to ALA treatment (**Table 2**). ALA significantly increased Ca content in roots but decreased it in leaves of Pb-stressed plants. The presence of Ca in roots may play a role in Pb accumulation within the cell wall and internal Pb detoxification (Antosiewicz, 2005). Additionally, ALA significantly reduced Pb concentration in both root and leaf tissues of Pb-stressed plants. Exogenous ALA has been shown to function as a metal chelator under heavy metal stress in various plant species (Sgherri et al., 2002; Turk et al., 2018; Yadav et al., 2022). Under environmental stress, the release of intracellular ROS is a common response in many plant species. This response is a key regulatory step in activating signal transduction pathways essential for defense mechanisms (Noctor et al., 2018). In this study, Pb-stressed exhibited elevated levels of H_2_O_2_ and increased rates of lipid peroxidation (MDA), consistent with previous findings (Qureshi et al., 2007; Thakur et al., 2017; Navabpour et al., 2020). Chloroplasts in the leaves are major sources of ROS in plant cells (Song et al., 2021), which may explain why H_2_O_2_ and MDA concentrations were higher in leaves than in roots in this study

Lipoxygenases (LOXs) are crucial enzymes that catalyze the conversion of polyunsaturated fatty acids into hydroperoxy fatty acids (linoleic and linolenic), playing a pivotal role in the biosynthesis of stress hormones, such as JA, MeJA and ABA (Viswanath et al., 2020; Shreya et al., 2022). In this study, Pb-stressed plants showed higher LOX activity in roots and leaves compared to unstressed plants, with a more pronounced increase in roots (**Figure 3C**). This elevation in LOX activity may be linked to increased lipid peroxidation rates in Pb-stressed plants. Conversely, ALA-treated plants exhibited a significant decrease in the LOX activity in both roots and leaves, likely due to ALA’s antioxidant properties, which reduce oxidative damage and lipid peroxidation (Navari-Izzo et al., 2002; Youssef et al., 2021; Yadav et al., 2022). Foliar application of ALA was more effective in inhibiting LOX activity in leaves than in roots, possibly due to direct contact with leaves or the predominant accumulation of Pb in roots.

Enhancing the ASA–GSH detoxification capacity is a key antioxidant defense strategy in plants under adverse conditions (Ding et al., 2009; Gill et al., 2013; Jahan et al., 2020). This cycle plays a pivotal role in reducing H_2_O_2_ overproduction and mitigating oxidative damage under stress conditions such as drought (Shan et al., 2018), salinity (Yan et al., 2018), alkalinity (Liu et al., 2015) and heavy metals (Singh et al., 2021; Bashir et al., 2022). ALA not only function as a metal chelator but also recycles antioxidants like tocopherols, GSH and ASA under heavy metal stress (Navari-Izzo et al., 2002). In this study, ALA-treated plants under Pb stress showed significant improvement in ASA and GSH levels, alongside a decline in DHA, GSSG, and their respective ratios (DHA/ASA and GSSG/GSH) in both leaves and roots (**Figures 4A–E**). These findings suggest that ALA helps protect reduced pools (ASA and GSH) and maintain redox homeostasis under Pb stress.

Pb can interfere with the activity of antioxidant enzymes involved in defensive mechanisms. In this study, the activities of SOD, POD, CAT, APX, GR and DHAR were investigated in tomato seedlings under Pb stress. Pb significantly reduced the activities of all studied enzymes except CAT and DHAR in both leaves and roots. The increased activities of CAT and DHAR suggest that H_2_O_2_ elimination by CAT and the restoration of AsA by DHAR are key defensive strategies against Pb-induced ROS. Exogenous ALA significantly enhanced the activities of all studied antioxidant enzymes except SOD in the roots. Previous studies have shown that ALA can modulate the activities of antioxidant enzymes in plants under stress conditions (Turk et al., 2018; Ramadan et al., 2022; Yadav et al., 2022; Daler and Kaya, 2024), indicating a potential relationship between ALA and the regulation of antioxidant systems (enzymatic and/or non-enzymatic).

The regression analysis between the major components of ascorbate- glutathione and Pb concentration revealed that AsA, GSH and GR were negatively correlated with the concentration of Pb. In contrast, DHA and GSSG were positively correlated with Pb concentration. These results imply that ALA can reduce the uptake of Pb by maintaining the redox balance through increasing the reduced components of ASA, GSH and enhancing the activity of GR. At the time there was an obvious decrease in the oxidized components of DHA and GSSG.

## 5 Conclusion

This investigation demonstrated the cumulative impacts of Pb toxicity, caused by irrigation with contaminated water, on tomato seedlings (**Figure 7**). Furthermore, it explored the effect of exogenous ALA in mitigating the harmful effects of Pb toxicity by modulating plant growth, photosynthetic pigments, nutrient homeostasis, ASA-GSH cycle and antioxidant enzymes activity. The results revealed that ALA enhanced redox status by stimulating the synthesis of AsA and GSH, while decreasing the oxidized forms (DHA and GSSG, respectively). Additionally, there was a significant inhibition in LOX activity, resulting in reduced oxidative damage and a significant decrease in lipid peroxidation. These responses were accompanied by noticeable modifications in the activity of antioxidant enzymes, including SOD, CAT, POD, APX, GR and DHAR. Overall, ALA efficiently alleviated Pb-induced phytotoxicity and improved Pb stress tolerance in tomato seedlings. Nevertheless, comprehensive molecular investigations are required to clarify the mechanisms in which ALA facilitates numerous defensive processes that improve plant tolerance to Pb toxicity and to determine dose-response relationships. In upcoming research, we plan to utilize advanced methodologies, including next-generation sequencing (NGS), in our laboratories to clarify the transcriptomic profile linked to ALA-mediated reduction of lead toxicity in Saudi Arabian agricultural soils and to identify genetic targets for the creation of resilient crop varieties.

## Data Availability

The original contributions presented in the study are included in the article/supplementary material. Further inquiries can be directed to the corresponding authors.
